# Iron, Tuberculosis, and Particle-Related Exposures Including Smoking

**DOI:** 10.3390/pathogens15090982

**Published:** 2026-09-16

**Authors:** Andrew J. Ghio, Edward R. Pennington, Jairus C. Pulczinski, Elizabeth D. Hilborn, Stephen L. Tilley

**Affiliations:** 1 US Environmental Protection Agency, Research Triangle Park, NC 27711, USA; ghio.andy@epa.gov (A.J.G.);; 2 College of Public Health, University of Arizona, Tucson, AZ 85721, USA; 3 Division of Pulmonary Diseases and Critical Care Medicine, Department of Medicine, University of North Carolina, Chapel Hill, NC 27599, USA

**Keywords:** iron, tuberculosis, smoking, air pollution, silicon dioxide

## Abstract

The availability of iron controls the abundance and diversity of life. Microbial pathways for iron acquisition account for their replication and virulence. The balance of interactions defining a microbial community can be altered by environmental stress. Decreased iron availability applies a selective pressure that results in increased competition for the metal. The complexation of the host metal by cigarette smoke and other particles induces a functional iron deficiency in the respiratory tract of the exposed individual. *M. tuberculosis* is at an advantage in environments of decreased iron availability after cigarette smoking and other particle exposures as it possesses numerous pathways for metal acquisition. Accordingly, smoking and other particle exposures (e.g., air pollution, burning of biomass, and silica) are associated with increased tuberculosis infections.

## 1. Introduction

Cigarette smoking is the leading cause of preventable deaths globally, with more than eight million each year from both direct use and secondhand exposure. In addition to deaths from cardiovascular disease, cancers, and chronic obstructive pulmonary disease (COPD), this excess mortality includes infections. Tuberculosis (TB) is one of the world’s deadliest infectious diseases, affecting individuals of all ages globally. In 2022, cases of TB globally numbered 10.6 million, resulting in 1.3 million deaths [1]. Not only has there been a strong correlation between smoking and TB since 1918, but other particle exposures are also associated with TB.

Increases in TB infection associated with smoking and other particle exposures can be explained in part by accompanying alterations in iron homeostasis. Specifically, the complexation of the metal at the particle surface is associated with a functional iron deficiency in the respiratory tract. *M. tuberculosis* has evolved to thrive in environments of low metal availability. Thus, particle-associated changes in iron homeostasis provide the microbe with a competitive advantage and allow it to proliferate in the exposed lung.

## 2. Methods

A review of the literature was performed using PubMed/MEDLINE, Web of Science, and TOXNET. All available years were included. There were no filters used for species, sex, age, or language. The first search string was (“Tuberculosis”[MeSH] and “Iron”[Majr]) and resulted in 129 articles. The second search string was (“Tuberculosis”[MeSH] and (“Smoking”[Mesh] or “Particulate matter”[MeSH] or “Environmental exposures”[MeSH] or “Silicon dioxide”[MeSH] or “Anthracosis”[Majr] or “Asbestos”[Majr]” or “Oxides”[MeSH] or “Minerals”[MeSH] or “Soil”[MeSH] or “Air pollutants”[MeSH] or “Wood”[MeSH])). This search resulted in 1421 articles.

## 3. Iron and Microbes

The adjustable oxidation–reduction potential and unique coordination chemistry led to the selection of iron for innumerable, critical cell functions. Consequently, iron is essential for almost every living system, and the availability of this metal controls the profusion and diversity of life [2]. However, despite being the fourth most abundant element on the Earth’s crust (~5%), iron concentrations are restricted in oxygen-containing environments at the physiological pH (~10^−18^ M). Accordingly, the quantities of the metal are below those necessary to support life in many environments (e.g., microbial growth requires ~10^−6^–10^−4^ M), obstructing cell replication and leading to regulated cell death [3].

In any environment, species and strains interact to obtain the required nutrients, with iron being predominant among these. Accordingly, microbes have developed abundant pathways for the acquisition of this critical metal (Table 1) [4,5,6,7,8]. The utilization of these pathways for iron acquisition governs the dynamics between microbes [9]. Within a polymicrobial community, pathways of iron acquisition applied by one microbe can be cooperative by providing the metal to others [10]. The production of siderophores (i.e., small, high-affinity iron chelators) is one such pathway that can lead to cooperative interactions [9,11]. The secretion of these chelators by one member of the community can positively influence the fitness of other species and strains through the provision of the requisite metal (“siderophore piracy”) [11]. Such cooperative interactions lead to dependencies and symbioses between microbes, contributing to stable communities [9]. However, these same pathways for iron acquisition can also lead to competitive interactions, with one microbe withholding the metal from others [9]. Accordingly, pathways for iron acquisition can both promote and impede microbe replication.

**Figure 1:**
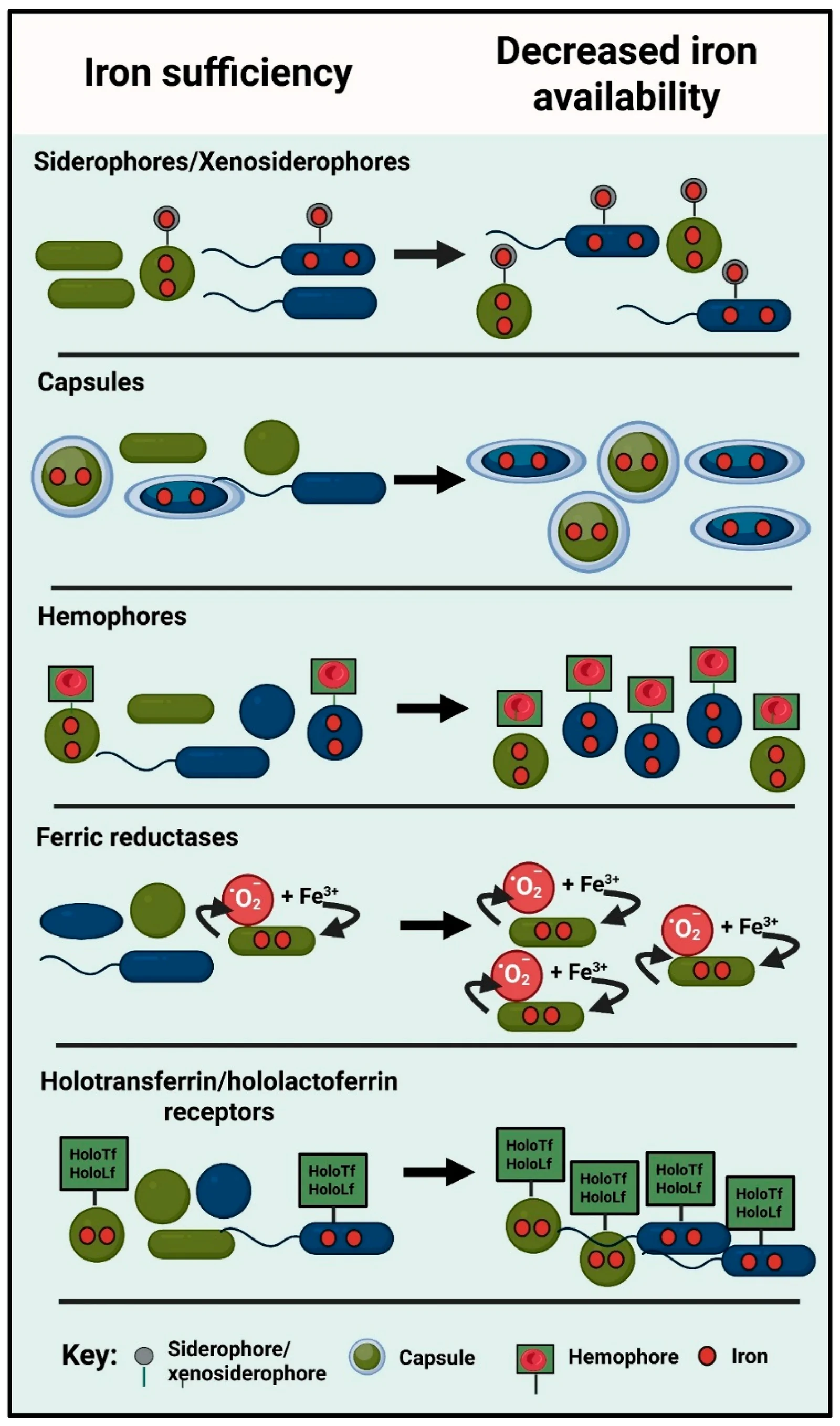
Pathways for metal acquisition commonly utilized by microbes. These can include siderophores, xenosiderophores, capsules, hemophores, reductases, and holotransferrin/hololactoferrin receptors. Such pathways determine competition for critical iron and, in an environment with decreased iron availability, subsequent microbial survival and duplication. Decreased iron availability in an environment applies a selective pressure, impacting microbial communities by compelling competition between them for the metal. With decreased metal availability, a microbe can demonstrate a superior capacity to acquire the metal and subsequently replicates. Diversity is lost when those microbials that can compete more effectively for requisite iron displace other species and replicate to dominate a population.

The balance of interactions included in cooperation and competition defines a community of microbials (e.g., a microbiota). These interactions, and subsequently the community itself, can be altered by environmental stress [12]. Decreased iron availability in an environment applies a selective pressure, impacting microbial communities by compelling competition between them for the metal. While all microbes have access to the metal, each has distinct requirements for iron and capacities for its acquisition. In response to the environmental stress of decreased iron availability, a microbe will exhibit significant phenotypic plasticity, dynamically modulating the expression of those pathways necessary for metal acquisition [13]. This adaptive approach optimizes energy expenditure and metal acquisition to confer an advantage to certain species and strains. Disparities in the capacity for iron acquisition will provide specific microbes with a competitive advantage in environments where the metal is a limited resource (Figure 1). Those that evolved efficient pathways (e.g., biosynthesis of secondary metabolites such as siderophores) will survive and replicate in iron-limited environments, where they can outcompete other microorganisms for metal acquisition [14]. By competing more effectively for requisite iron, the microbe displaces other species and grows to dominate a community. Thus, the competitive success of a microorganism can reflect the ability of the microbe to monopolize iron availability [9].

**Figure 2:**
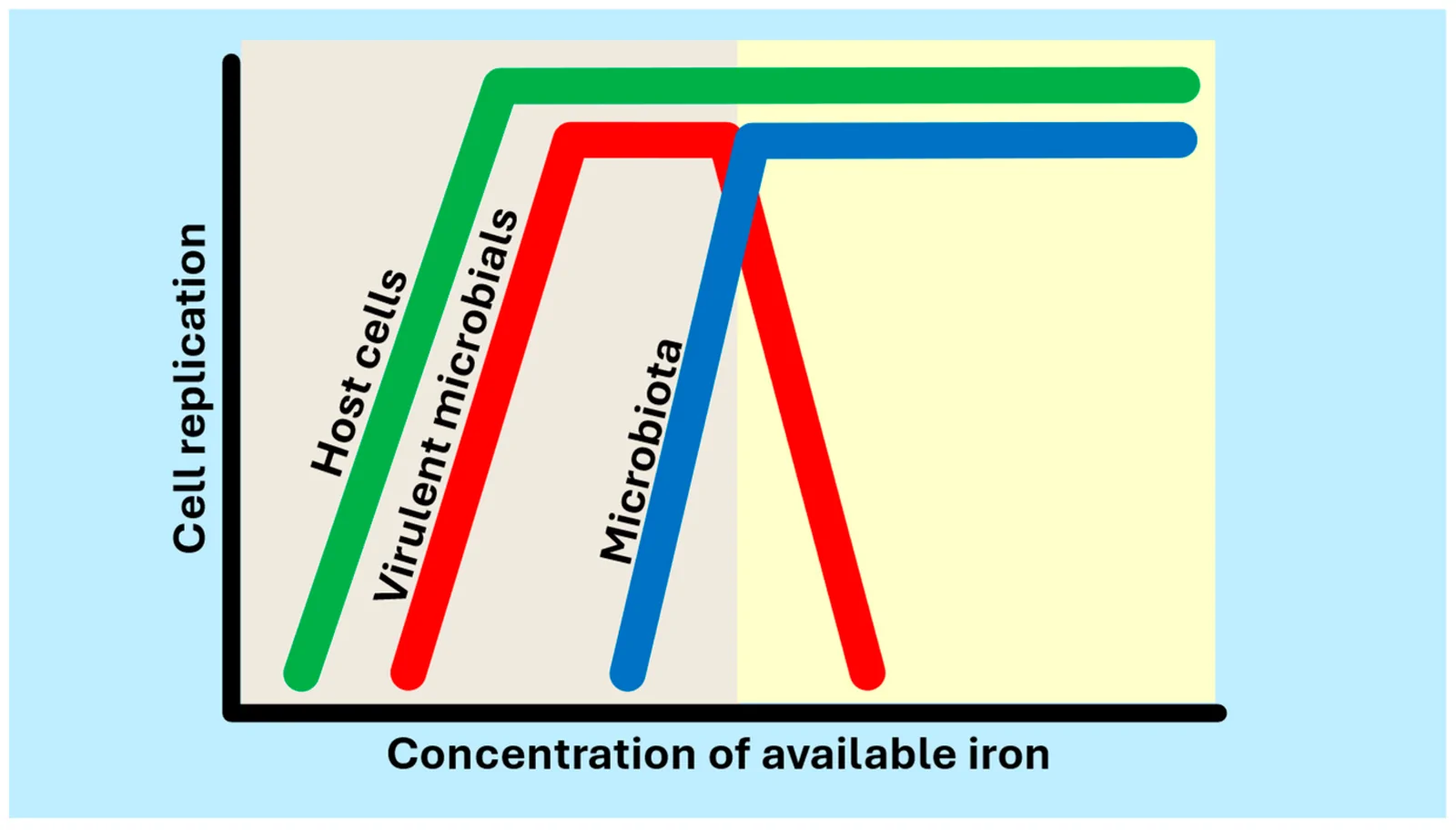
The relationship between iron availability and cell replication. With iron sufficiency (yellow background), there can be cooperation among members of the polymicrobial community. With decreased availability of the metal, those microbes with efficient iron acquisition pathways will be selected to replicate. The provision of iron might benefit the microbiota by preventing competition and the development of pathogenic and virulent microbials in the lung, depending on the extent of limitation. However, if virulent microbes are included in the microbiota (beige background), the provision of the metal would likely be followed by their preferential replication.

By allowing for the replication of microbes with a greater capacity for metal acquisition relative to others, limited iron availability selects specific microbes, leading to lower diversity (i.e., the number and distribution of different types of microorganisms). Accordingly, a microbiota can be dynamically modulated by the available iron [15]. Moreover, competition for iron can disrupt stable communities that were previously characterized by cooperative interactions. The subsequent changes in the structure of a microbial community impacted by limited iron concentrations can be protracted [16]. Dietary iron depletion imprints low diversity in the microbiota that is sometimes not easily recovered [17]. Those species that are more readily lost have the greatest dependence on iron for survival and the weakest ability to compete successfully, and they can belong to common phyla in a microbiota (i.e., commensals). However, the reversal of iron deficiency can be associated with the recovery of an indigenous microbial population’s diversity to normal levels and decrease the development of pathogens in an environment (Figure 2) [18].

## 4. Iron and Microbes in the Human

Pathways for iron acquisition also define the interactions between microbes and human cells. These can similarly be both cooperative and competitive. Microbial virulence in the human can be defined by an increased capacity to successfully acquire the metal and replicate in the iron-limited environment of a host tissue [9,19]. With a microbe competing effectively for requisite iron, virulent species and strains displace both other microorganisms and host cells to dominate an environment. Iron availability in the human is a major regulator impacting microbial replication [20]. Decreased iron availability in an environment selects for specific microbes [21]. While microbes lacking the capacity to acquire iron in a metal-deficient environment will demonstrate reduced growth, those that can utilize efficient pathways to procure the metal have an advantage and replicate, affecting human health [21].

Microbial siderophores act directly on host cells to not only procure the required metal but also stimulate the production and release of cytokines (e.g., interleukin (IL)-6 and IL-8), which initiates and coordinates the inflammatory response [22]. Similarly, microbes may use toxins to bind and transport iron, also leading to an inflammatory response in the host (e.g., the toxin domoic acid, an iron chelator produced by a microbe to facilitate iron import, is associated with both shellfish poisoning and host mortality) [23]. Subsequently, in a human host, decreased iron availability in an environment selects for virulent species and initiates human morbidity (e.g., inflammation) and mortality associated with microbial exposure.

For the human host, a major defense against infection is the withholding of iron to prevent microbial replication (e.g., nutritional immunity). This is included in the acute phase of the inflammatory response [24]. Almost all human iron is intracellular, with most of it being complexed within the porphyrin ring of heme as a cofactor of hemoglobin/myoglobin and sequestered within the iron storage protein ferritin. The small quantity of extracellular iron is bound by the circulating glycoprotein transferrin, which strongly complexes the metal (K_a_ ~ 10^36^ M^−1^). Humans are therefore almost devoid of available iron, ensuring that microbes encounter a period of iron starvation upon initial access. While microbes have developed numerous pathways for iron acquisition from the host, human cells have evolved pathways for metal procurement and sequestration that are frequently indistinguishable from those of the microbes (Table 2) [25]. By increasing their iron acquisition, the host cells compete with the microbe for survival and limit access to the metal, creating an environment that is unfavorable for microbial replication. This effort supports host cell survival and proliferation. Interactions between host cells and microbes in obtaining the required metal become complicated with the development of ancillary pathways for microbial iron acquisition. Through such pathways, pathogens are further selected by host iron limitation as the microbes attempt to outmaneuver the host cells (e.g., the expression and release of “stealth” siderophores not recognized by host lipocalins and the expression of competitive antagonists that bind lipocalins in lieu of siderophores) [26].

## 5. Iron and TB

Utilizing a plethora of pathways, *M. tuberculosis* is exceptionally proficient at acquiring host iron from cells in the respiratory tract. With metal limitation in the host, *M. tuberculosis* adapts to survive by triggering a coordinated transcriptional response, leading to the upregulation of numerous iron acquisition pathways, which accordingly function as virulence factors [27,28]. *M. tuberculosis* can produce two siderophores: mycobactin and carboxymycobactin. Mycobactin belongs to the hydroxamate class of siderophores and is lipophilic, while carboxymycobactin is a mixed-type siderophore containing both hydroxamate and catecholate groups. Mycobactin is cell wall-associated, while carboxymycobactin is a soluble, secreted compound that can access extracellular iron. Iron is efficiently released from mycobactins through either the degradation of the chelator or iron reduction. Highlighting the importance of these factors, a loss of siderophore function attenuates *M. tuberculosis* virulence. *M. tuberculosis* also utilizes xenosiderophores, internalizes holotransferrin and hololactoferrin, possesses at least two heme uptake pathways, expresses toxins that contribute to metal acquisition (e.g., hemolysins that can lyse erythrocyte cells, releasing hemoglobin and creating a pool of labile heme, which is bound by hemophores), and encodes two iron storage proteins—a bacterioferritin and a ferritin-like protein. The large number of efficient pathways for iron acquisition utilized by *M. tuberculosis* supports the enormous capacity of this specific microbe for virulence (i.e., to thrive in an iron-limited environment).

A relationship between TB and iron availability has been suggested by historical observations. The increased risk of Native American populations for TB, beginning in the 15th century and continuing until recently, has been accompanied by widespread iron deficiency, due in part to a dietary reliance on maize [29]. In the 1700s, TB was called the “white plague”, as affected individuals demonstrated a pale complexion, likely reflecting anemia, which was considered to enhance the physical beauty of those dying with the disease. Historically, food insecurity, malnutrition, and starvation, exemplified by the Dutch famine of 1944–1945 (“Winter of Hunger”), have increased the risk for TB and are recognized as being associated with iron deficiency, as well as being the most frequent cause of immune deficiency [30,31,32].

An investigation focused on better understanding the relationship between metal availability and TB has confirmed that there is a higher incidence of TB among those diagnosed with iron deficiency anemia, reflecting the limited quantities of available metal in the host [27]. Studies have demonstrated that there is greater lung injury and a worse prognosis among TB patients with anemia [28]. In one study, almost half of all sputum-positive pulmonary TB patients were anemic, and, among these, 79.1% had an iron deficiency [33]. In another study, 90% of patients with TB had either anemia of chronic disease or iron deficiency anemia [34]. Women are at a greater risk of developing TB, including active infection, during times at which iron deficiency is expected (e.g., pregnancy and postpartum) [35,36]. Iron deficiency with and without anemia has been associated with a two- to three-fold increased risk of death associated with TB [37]. With iron deficiency and iron deficiency anemia, the numerous pathways for metal acquisition confer pathogenicity and virulence upon *M. tuberculosis*, which displaces both other microbes and host cells.

The human host responds to the challenge of *M. tuberculosis* by sequestering its own iron, thus limiting its availability. Following the phagocytosis of the slow-growing, acid-fast bacterium, cytoplasmic iron is actively exported by macrophages and derived cells. The expression of natural resistance-associated macrophage protein 1 (Nramp1 or solute carrier family 11A1), a late endosomal membrane transporter found in macrophages and neutrophils, is increased to transport iron out of the phagolysosome and away from the mycobacteria [38]. The host phagolysosome will subsequently have diminished concentrations of the metal to support *M. tuberculosis* growth. In humans, Nramp1 expression confers host resistance against intracellular pathogens, which survive within phagosomes, including mycobacteria. The overexpression of ferroportin-1 by the host—the sole iron exporter—similarly decreases cell iron availability to *M. tuberculosis* and impairs its survival, validating selective metal deprivation as an anti-mycobacterial approach [39]. Cell production of the iron-sequestering protein lipocalin-2 also increases with infection. These and other host pathways for iron acquisition augment the capacity for the host to eradicate *M. tuberculosis*. A transcriptional analysis has indicated that tuberculous granulomas express high levels of iron uptake genes (e.g., heme-binding proteins, hemoglobin receptors, haptoglobin, hemopexin, heme oxygenase, transferrin receptor-1, lactoferrin, lipocalin, calprotectin, and ferritin), reflecting an attempt by the host to restrict metal availability to the microbe [40].

## 6. Iron and Smoking

Smoking one cigarette exposes the human respiratory tract to between 15,000 and 40,000 µg of particles [41]. The deposition fraction of cigarette smoke particles (CSPs), with a mean diameter of about 0.2–0.5 µm, in the lung is 70–90%. Soluble components of CSPs can be transported to the blood. Mucociliary clearance transports the insoluble components of CSPs to the gastrointestinal tract and lymph nodes; this is slowest from the alveolar region, where a significant portion of clearance is dependent on macrophage function. Therefore, the lungs of ever-smokers retain enormous numbers of particles.

CSPs include humic-like substances (HULIS; approximately 7–10%), which are complex, organic, macromolecular compounds. Oxygen-containing functional groups within HULIS (e.g., phenolates/catecholates and carboxylates) react with metal cations to form coordination complexes, and, among these, ferric ion is kinetically the most favored [42]. Subsequently, exposure to CSPs, with their included HULIS, results in retention and cell exposure to an inanimate surface composed of functional groups with a capacity to complex metals and alter iron homeostasis. The complexation of host cell iron by the particle surface can be equivalent to the sequestration of the metal by microbial siderophores. Comparably to its response to virulent microbes, the host cell must compete with the particle for its iron. With smoking, iron-laden macrophages (i.e., sideromacrophages) are observed in the respiratory tract, reflecting the capacity of CSPs to complex and sequester host cell metal [41]. Given the ability of CSPs to complex and sequester iron, the concentration of available iron in the lung will be diminished. This has been investigated using exhaled breath condensate, which revealed decreased iron concentrations in the respiratory tracts (i.e., the epithelial lining fluid) of smokers [43]. As CSPs’ complexation of host iron leads to a functional iron deficiency, the cell attempts to reverse the loss of requisite iron via the increased expression of proteins involved in metal import (e.g., transferrin receptor and DMT1) [44]. Compared to non-smokers’ lungs, both transferrin and transferrin receptor expression in cells and fluids can be higher in those with smoking-related disease, reflecting decreased metal availability. The import of iron frequently requires ferrireduction (e.g., superoxide generation), which is also increased in host cells after CSP exposure, again reflecting decreased metal availability. In the smoker, cells exposed to CSPs subsequently increase iron import due to decreased metal availability. If either enough host iron is complexed by the particle or the cell response to increase the metal is inadequate, function and survival will be compromised.

Reflecting metal limitation after smoking, iron deficiency is common in these populations [45]. Anemia is more frequently observed in both smokers and populations with smoking-related disease (e.g., patients with COPD). The two primary types of anemia seen in smoking-related disease are anemia of chronic disease and iron deficiency anemia; both are associated with altered iron homeostasis. Smoking can impact iron deficiency anemia [46]. During pregnancy, there are lower concentrations of ferritin in both the placenta and umbilical cord blood, with smoking by the mother leading to diminished metal availability. A regression analysis showed that tobacco use was independently associated with maternal iron deficiency anemia in the newborn. Finally, environmental tobacco smoke can be associated with anemia, which is often related to iron deficiency.

## 7. TB and Smoking

**Figure 3:**
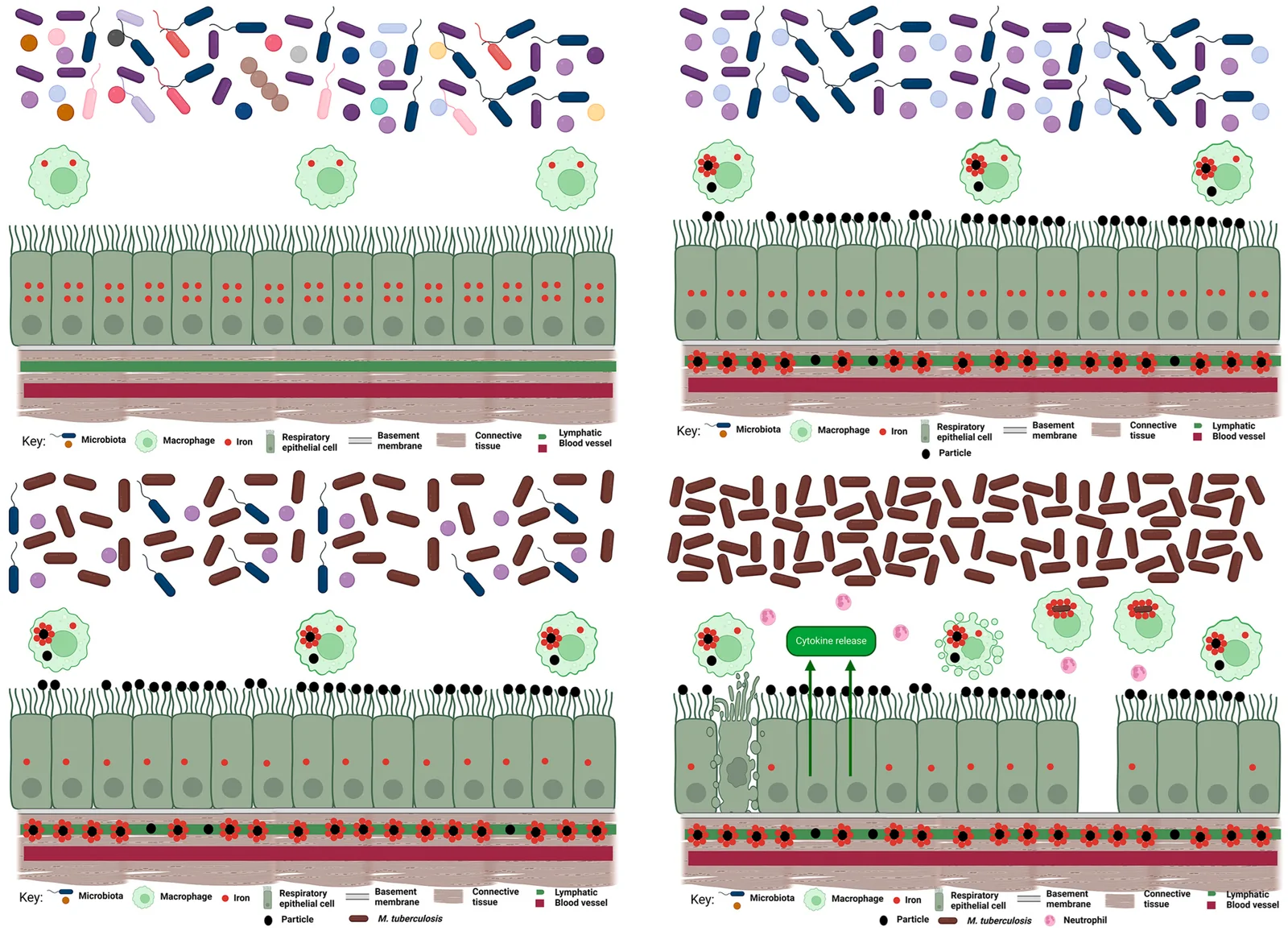
Smoking alters host iron homeostasis, decreases metal availability, and selects virulent microbials for replication. Iron homeostasis in the airways determines that some level of metal is available for both host cell and microbiota function and replication (**top left**). The iron in the cells of the airway is complexed by cigarette smoke particles, and availability to both the host cells and microbiota is decreased; diversity is lost among the microbes (**top right**). With the introduction of a virulent microbe with efficient pathways for iron acquisition (e.g., *M. tuberculosis*), the environment supports its replication (**bottom left**). Finally, the virulent microbe dominates, and its utilization of host cell metal initiates inflammatory pathways (e.g., siderophores interacting with macrophages and airway epithelial cells) (**bottom right**).

The microbiota in the lungs, which participates in the interactions between environmental exposures and respiratory health, is altered by smoking, with the selection of virulent microorganisms and decrements in the diversity of microbial assemblies comparable to the diminished availability of iron [47,48]. Decreased iron availability in the smoker’s respiratory tract selects *M. tuberculosis* to express additional pathways of iron acquisition, allowing access to the metal, which is required for replication and virulence (Figure 3) [49]. Greater than 10% of all TB cases in the world are attributed to smoking, which increases the risk for TB, including active and latent infections and disease progression, relapse, and mortality [50,51,52]. Smokers have higher rates of both tuberculin skin test reactivity/conversion and active TB infection [53]. Indeed, studies have shown dose–response relationships between (1) the duration of smoking and the number of cigarettes smoked per day and (2) the rate of TB infection observed [54]. The death rate from TB is about four times higher in those who are ever-smokers relative to those who have never smoked. The diagnosis of a smoking-related disease (e.g., COPD or idiopathic pulmonary fibrosis) increases the risk for TB, attributed to exposure to CSPs and decreased iron availability [55]. In addition to active adult smokers, those who experience secondhand smoke, also referred to as passive smoking or environmental tobacco smoke exposure, are at an elevated risk for latent and active TB [50,56,57]. With such exposure, *M. tuberculosis* infection increases with the number of smokers in the home.

## 8. TB and Particle Exposures Other than Smoking

Particles other than CSPs also complex metals via surface functional groups (e.g., silicates used as water filters) and impact cell metal homeostasis, with a resultant functional cell iron deficiency [58]. Decreased iron availability is associated with an increased risk of TB infection, similarly to smoking.

Numerous carbonaceous particles (e.g., diesel exhaust and air pollution particles) can include HULIS (approximately 5–10%) [59]. However, carbonaceous particles contain surface components other than HULIS, which present multiple functional groups with the ability to complex metals, including iron (e.g., phenolates/catecholates, carboxylates, alcohols, diols, epoxides, ethers, aldehydes, ketones, and esters). Comparably to smoking, the inhalation of these particles is associated with an increased risk for TB infection. Air pollution, both ambient and household, is a significant global public health issue associated with millions of premature deaths worldwide annually, and this includes mortality from infections. Exposure to particulate matter (measured as PM_2.5_, PM_10_, and black carbon) is associated with an increased incidence of TB infection [60,61,62]. Models demonstrate a significant correlation among smear-positive status, reflecting TB infection, and residential exposure to PM_2.5_ [63]. Wildfire-associated ambient air pollution is associated with an increased risk of active TB diagnosis [64]. Exposure to the burning of biomass also increases the risk for TB; this is especially true in women [51,65,66]. Similarly, agricultural work is associated with significant exposure to particles and increases mortality from TB infection [67].

Inorganic particles (e.g., silica, silicates, mineral oxides, and desert dust) also include oxygen-containing functional groups on their surfaces (e.g., silanol groups in silica and silicates) and introduce an electronegative interface into a cell with their exposure. Complex formation by such polyanionic surfaces prefers iron because of its electropositivity [68]. Accordingly, exposure to inorganic particles also leads to functional metal deficiency, which may increase the risk for TB infection. In particular, inorganic particle exposure during mining, tunneling, and foundry work has been shown to increase the risk for TB infection [69]. Infections associated with silica exposure remain a clinical and significant public health problem [70,71]. In the past, TB infections have been observed to be increased among workers exposed to asbestosis, kaolin, fluorspar, and talc [72,73,74,75]. Exposure to inorganic particles (which can include silica and silicates) in desert dust storms similarly increases the risk for TB [76]. Those with particle-related disease, both malignant and non-malignant, can be at an increased risk for TB infection [77]. The increase in mycobacterial infections following silica and silicate exposures is not restricted to TB but includes atypical mycobacteria [78,79,80].

Peaking in the 1700s and the 1800s and receding slowly afterwards, the incidence and prevalence of TB infection corresponded to changes in particle exposure associated with industrialization (e.g., exposure to steam engines, blast furnaces, and mines) and urbanization (e.g., exposure to coal-burning stoves used for heat), which resulted in smogs, such as those in London during the Great Smog of London, also known as “The Big Smoke” (December, 1952). Historical statistics on coal consumption and TB disease in Canada, USA, and China are correlated positively [81]. While a therapeutic intervention was not introduced until 1943 (i.e., with the discovery of streptomycin), there was a slow decline in TB deaths starting in the 1860s–1870s. This has been attributed to general improvements in the living standards of populations during the second half of the 19th and the first half of the 20th century, including the control of exposure to particles generated at work sites and during heating and food preparation.

## 9. Conclusions

In the lung, those microbes with efficient pathways for metal acquisition are selected by iron limitation to dominate a microbiota. Accordingly, smoking and other particle exposures, which complex and decrease available iron, are associated with increased TB infections. The diminished iron concentrations following particle exposure select *M. tuberculosis* to outcompete other members of the microbiota in the lung.

## Figures and Tables

**Table 1:** Pathways for iron acquisition that are frequently utilized by microbes.

Pathway	Description
Siderophores	• Small-molecular-weight chelators with K_a_ up to 10^52^ M^−1^. • Between 2 and 6 O- and N- electron donor atoms included in catecholate, hydroxamate, carboxylate, phenolate, and mixed functional groups. • Resultant hexadentate iron complex is recognized by surface-displayed receptors and internalized by active transport. • Liberation of the metal by either reduction or proteolytic cleavage.
Xenosiderophores	• Iron chelator used by a microbe other than that which created it. • No energetic requirement for de novo synthesis. • Membrane transporters are not limited to one siderophore.
Low-affinity ligands	• Ligands maintain metal solubility to increase availability. • Prominent among these are substances with multiple functional groups with low affinities for cationic metals (e.g., carboxylates such as sugar acids). • Microbes produce and couple their release with cognate receptors, enhancing metal acquisition.
Capsules	• Polysaccharides incorporated into a capsule, which functions as a reservoir for the metal (e.g., alginate).
Biofilms	• Polysaccharides define mucilage in biofilms, which binds iron via carboxyl, carbonyl, and sulfate functional groups.
Hemophores	• Directly bind heme, the most abundant iron source in many environments.
Ferric iron reductases	• Reduction of ferric ion not only to access insoluble precipitates but also to non-specifically liberate iron from metal complexes.
Receptors for iron-binding proteins	• Membrane receptors bind holotransferrin and hololactoferrin, followed by metal complex internalization.
Toxins	• Secondary metabolites that can bind iron to alter homeostasis and increase availability (e.g., microcystins and domoic acid).

**Table 2:** Pathways for iron acquisition that are frequently utilized by humans.

Pathway	Description
Siderophilins	• Release of apolactoferrin (e.g., from secondary granules in leukocytes) complexes and transports the metal (e.g., to the reticuloendothelial system).
Siderocalins	• Release of lipocalin-2 sequesters catecholate-type siderophores with its complexed iron.
Hemophores	• Hemoglobin or heme released during the erythrocyte cycle is captured by haptoglobin and hemopexin, respectively, and delivered with the metal (e.g., to the reticuloendothelial system).
Ferric iron reductases	• Increased activity of oxidoreductases, which function as ferric reductases to facilitate the transport of iron across the host cell membrane. • Participate in macrophages, actively “pumping out” iron from a phagosome.
Receptors for iron-binding proteins	• Increased siderophilin, haptoglobin, and hemopexin receptors.
Metabolic adaptation	• Upregulation of glycolysis and pentose phosphate pathways.
Hepcidin	• Hepatic hormone blocks metal uptake by the enterocyte. • Binds and degrades the iron exporter ferroportin-1, sequestering iron in macrophages. • Decreases iron release into plasma.
Ferritin	• Greater sequestration of transferrin- and lactoferrin-bound iron by macrophages with storage in ferritin.
Release of cytokines	• Diverts transport of iron, leading to its retention (e.g., in the reticuloendothelial system).

## Data Availability

No new data were created or analyzed in this study. Data sharing is not applicable.

## References

[1] Feldman C., Theron A.J., Cholo M.C., Anderson R. (2024). Cigarette Smoking as a Risk Factor for Tuberculosis in Adults: Epidemiology and Aspects of Disease Pathogenesis. Pathogens.

[2] Ryan-Keogh T.J., Thomalla S.J., Monteiro P.M.S., Tagliabue A. (2023). Multidecadal trend of increasing iron stress in Southern Ocean phytoplankton. Science.

[3] Haq R.U., Wereley J.P., Chitambar C.R. (1995). Induction of apoptosis by iron deprivation in human leukemic CCRF-CEM cells. Exp. Hematol..

[4] Rose A.L., Salmon T.P., Lukondeh T., Neilan B.A., Waite T.D. (2005). Use of superoxide as an electron shuttle for iron acquisition by the marine cyanobacterium *Lyngbya majuscula*. Environ. Sci. Technol..

[5] Caza M., Kronstad J.W. (2013). Shared and distinct mechanisms of iron acquisition by bacterial and fungal pathogens of humans. Front. Cell. Infect. Microbiol..

[6] Bairwa G., Jung W.H., Kronstad J.W. (2017). Iron acquisition in fungal pathogens of humans. Metallomics.

[7] Chan D.C.K., Burrows L.L. (2023). Pseudomonas aeruginosa FpvB Is a High-Affinity Transporter for Xenosiderophores Ferrichrome and Ferrioxamine B. mBio.

[8] Brunson D.N., Manzer H., Smith A.B., Zackular J.P., Kitten T., Lemos J.A. (2025). Characterization of a heme-degrading enzyme that mediates fitness and pathogenicity in *Enterococcus faecalis*. mBio.

[9] Kramer J., Ozkaya O., Kummerli R. (2020). Bacterial siderophores in community and host interactions. Nat. Rev. Microbiol..

[10] Figueiredo A.R.T., Özkaya Ö., Kümmerli R., Kramer J. (2022). Siderophores drive invasion dynamics in bacterial communities through their dual role as public good versus public bad. Ecol. Lett..

[11] Galdino A.C.M., Vaillancourt M., Celedonio D., Huse K., Doi Y., Lee J.S., Jorth P. (2024). Siderophores promote cooperative interspecies and intraspecies cross-protection against antibiotics in vitro. Nat. Microbiol..

[12] Jin Z., Li J., Ni L., Zhang R., Xia A., Jin F. (2018). Conditional privatization of a public siderophore enables *Pseudomonas aeruginosa* to resist cheater invasion. Nat. Commun..

[13] Joshi H., Khan A. (2024). Competition-driven phenotypic plasticity in Iron acquisition and aromatic utilization confers a fitness advantage to *Pseudomonas putida* in an Iron-limited rhizospheric environment. World J. Microbiol. Biotechnol..

[14] Lee N., Kim W., Chung J., Lee Y., Cho S., Jang K.S., Kim S.C., Palsson B., Cho B.K. (2020). Iron competition triggers antibiotic biosynthesis in *Streptomyces coelicolor* during coculture with *Myxococcus xanthus*. ISME J..

[15] Yilmaz B., Li H. (2018). Gut Microbiota and Iron: The Crucial Actors in Health and Disease. Pharmaceuticals.

[16] McClorry S., Zavaleta N., Llanos A., Casapía M., Lönnerdal B., Slupsky C.M. (2018). Anemia in infancy is associated with alterations in systemic metabolism and microbial structure and function in a sex-specific manner: An observational study. Am. J. Clin. Nutr..

[17] Pereira D.I., Aslam M.F., Frazer D.M., Schmidt A., Walton G.E., McCartney A.L., Gibson G.R., Anderson G.J., Powell J.J. (2015). Dietary iron depletion at weaning imprints low microbiome diversity and this is not recovered with oral Nano Fe(III). MicrobiologyOpen.

[18] Seo H., Yoon S.Y., Ul-Haq A., Jo S., Kim S., Rahim M.A., Park H.A., Ghorbanian F., Kim M.J., Lee M.Y. (2023). The Effects of Iron Deficiency on the Gut Microbiota in Women of Childbearing Age. Nutrients.

[19] Sheldon J.R., Laakso H.A., Heinrichs D.E. (2016). Iron Acquisition Strategies of Bacterial Pathogens. Microbiol. Spectr..

[20] Lewis J.P., Gui Q. (2023). Iron Deficiency Modulates Metabolic Landscape of Bacteroidetes Promoting Its Resilience during Inflammation. Microbiol. Spectr..

[21] Sun B., Tan B., Zhang P., Zhu L., Wei H., Huang T., Li C., Yang W. (2024). Iron deficiency anemia: A critical review on iron absorption, supplementation and its influence on gut microbiota. Food Funct..

[22] Holden V.I., Lenio S., Kuick R., Ramakrishnan S.K., Shah Y.M., Bachman M.A. (2014). Bacterial siderophores that evade or overwhelm lipocalin 2 induce hypoxia inducible factor 1α and proinflammatory cytokine secretion in cultured respiratory epithelial cells. Infect. Immun..

[23] Brunson J.K., Thukral M., Ryan J.P., Anderson C.R., Kolody B.C., James C.C., Chavez F.P., Leaw C.P., Rabines A.J., Venepally P. (2024). Molecular forecasting of domoic acid during a pervasive toxic diatom bloom. Proc. Natl. Acad. Sci. USA.

[24] de Brito E.D.C.A., Siqueira I.V., Venturini J., Félix V.L.T., Dos Santos A.O.G.M., Mendes R.P., Weber S.S., Paniago A.M.M. (2023). Iron metabolism disorders of patients with chronic paracoccidioidomycosis. PLoS ONE.

[25] Partridge J., Wallace D.F., Raja K.B., Dooley J.S., Walker A.P. (1998). Monocyte-macrophage ferric reductase activity is inhibited by iron and stimulated by cellular differentiation. Biochem. J..

[26] Golonka R., Yeoh B.S., Vijay-Kumar M. (2019). The Iron Tug-of-War between Bacterial Siderophores and Innate Immunity. J. Innate Immun..

[27] Chu K.A., Hsu C.H., Lin M.C., Chu Y.H., Hung Y.M., Wei J.C. (2019). Association of iron deficiency anemia with tuberculosis in Taiwan: A nationwide population-based study. PLoS ONE.

[28] Luo M., Liu M., Wu X., Wu Y., Yang H., Qin L., Yu F., Hu Y., Liu Z. (2022). Impact of anemia on prognosis in tuberculosis patients. Ann. Transl. Med..

[29] El-Najjar M.Y., Andrews J., Moore J.G., Bragg D.G. (1982). Iron deficiency anemia in two prehistoric American Indian skeletons: A dietary hypothesis. Plains Anthropol..

[30] Van Cleeff M.R.A., Hueting E., Dessing A., Murray J.F., Loddenkemper R. (2018). Tuberculosis in the Netherlands before, during, and after World War II. Tuberculosis and War. Lessons Learned from World War II.

[31] Franco J.V., Bongaerts B., Metzendorf M.I., Risso A., Guo Y., Peña Silva L., Boeckmann M., Schlesinger S., Damen J.A., Richter B. (2024). Undernutrition as a risk factor for tuberculosis disease. Cochrane Database Syst. Rev..

[32] Schwenk A., Macallan D.C. (2000). Tuberculosis, malnutrition and wasting. Curr. Opin. Clin. Nutr. Metab. Care.

[33] Metanat M., Mashhadi M.A., Alavi-Naini R., Rezaie-Kahkhaie L., Sepehri-Rad N., Afshari M. (2020). The Prevalence of Absolute and Functional Iron Deficiency Anemia in New Cases of Smear-positive Pulmonary Tuberculosis and Their Sputum Conversion Rate at the End of Intensive Tuberculosis Treatment Phase. Prague Med. Rep..

[34] Bashir B.A., Mohamed H.M., Hassan M.M., Ali W.Y., Moglad E., Hussain M.A., Osman W., Alsiyud D.F., Mohamed G.A., Ibrahim S.R.M. (2025). Role of Iron Indices in Anemia in Patients with Pulmonary Tuberculosis. Interdiscip. Perspect. Infect. Dis..

[35] Zenner D., Kruijshaar M.E., Andrews N., Abubakar I. (2012). Risk of tuberculosis in pregnancy: A national, primary care-based cohort and self-controlled case series study. Am. J. Respir. Crit. Care Med..

[36] Mathad J.S., Yadav S., Vaidyanathan A., Gupta A., LaCourse S.M. (2022). Tuberculosis Infection in Pregnant People: Current Practices and Research Priorities. Pathogens.

[37] Isanaka S., Mugusi F., Urassa W., Willett W.C., Bosch R.J., Villamor E., Spiegelman D., Duggan C., Fawzi W.W. (2012). Iron deficiency and anemia predict mortality in patients with tuberculosis. J. Nutr..

[38] Rodrigues P.N., Gomes S.S., Neves J.V., Gomes-Pereira S., Correia-Neves M., Nunes-Alves C., Stolte J., Sanchez M., Appelberg R., Muckenthaler M.U. (2011). Mycobacteria-induced anaemia revisited: A molecular approach reveals the involvement of NRAMP1 and lipocalin-2, but not of hepcidin. Immunobiology.

[39] Baker M.A., Wilson D., Wallengren K., Sandgren A., Iartchouk O., Broodie N., Goonesekera S.D., Sabeti P.C., Murray M.B. (2012). Polymorphisms in the gene that encodes the iron transport protein ferroportin 1 influence susceptibility to tuberculosis. J. Infect. Dis..

[40] Kurthkoti K., Amin H., Marakalala M.J., Ghanny S., Subbian S., Sakatos A., Livny J., Fortune S.M., Berney M., Rodriguez G.M. (2017). The Capacity of Mycobacterium tuberculosis to Survive Iron Starvation Might Enable It to Persist in Iron-Deprived Microenvironments of Human Granulomas. mBio.

[41] Ghio A.J., Hilborn E.D., Stonehuerner J.G., Dailey L.A., Carter J.D., Richards J.H., Crissman K.M., Foronjy R.F., Uyeminami D.L., Pinkerton K.E. (2008). Particulate matter in cigarette smoke alters iron homeostasis to produce a biological effect. Am. J. Respir. Crit. Care Med..

[42] Ghio A.J., Stonehuerner J., Quigley D.R. (1994). Humic-like substances in cigarette smoke condensate and lung tissue of smokers. Am. J. Physiol..

[43] Ghio A.J., Soukup J.M., McGee J., Madden M.C., Esther C.R. (2018). Iron concentration in exhaled breath condensate decreases in ever-smokers and COPD patients. J. Breath Res..

[44] Mayo J.J., Kohlhepp P., Zhang D., Winzerling J.J. (2004). Effects of sham air and cigarette smoke on A549 lung cells: Implications for iron-mediated oxidative damage. Am. J. Physiol. Lung Cell. Mol. Physiol..

[45] Vasquez A., Logomarsino J.V. (2016). Anemia in chronic obstructive pulmonary disease and the potential role of iron deficiency. COPD J. Chronic Obstr. Pulm. Dis..

[46] Vivek A., Kaushik R.M., Kaushik R. (2023). Tobacco smoking-related risk for iron deficiency anemia: A case-control study. J. Addict. Dis..

[47] Kumar P.S., Matthews C.R., Joshi V., de Jager M., Aspiras M. (2011). Tobacco smoking affects bacterial acquisition and colonization in oral biofilms. Infect. Immun..

[48] Lacoma A., Edwards A.M., Young B.C., Domínguez J., Prat C., Laabei M. (2019). Cigarette smoke exposure redirects Staphylococcus aureus to a virulence profile associated with persistent infection. Sci. Rep..

[49] Cronjé L., Edmondson N., Eisenach K.D., Bornman L. (2005). Iron and iron chelating agents modulate Mycobacterium tuberculosis growth and monocyte-macrophage viability and effector functions. FEMS Immunol. Med. Microbiol..

[50] Pai M., Mohan A., Dheda K., Leung C.C., Yew W.W., Christopher D.J., Sharma S.K. (2007). Lethal interaction: The colliding epidemics of tobacco and tuberculosis. Expert Rev. Anti Infect. Ther..

[51] Lin H.H., Ezzati M., Murray M. (2007). Tobacco smoke, indoor air pollution and tuberculosis: A systematic review and meta-analysis. PLoS Med..

[52] Quan D.H., Kwong A.J., Hansbro P.M., Britton W.J. (2022). No smoke without fire: The impact of cigarette smoking on the immune control of tuberculosis. Eur. Respir. Rev..

[53] Nisar M., Williams C.S., Ashby D., Davies P.D. (1993). Tuberculin testing in residential homes for the elderly. Thorax.

[54] Park S.W., Song J.W., Shim T.S., Park M.S., Lee H.L., Uh S.T., Park C.S., Kim D.S. (2012). Mycobacterial pulmonary infections in patients with idiopathic pulmonary fibrosis. J. Korean Med. Sci..

[55] Liao K.M., Kuo L.T., Lu H.Y. (2025). The Risk of Tuberculosis in Chronic Obstructive Pulmonary Disease Across Different Comorbidities. J. Clin. Med. Res..

[56] du Preez K., Mandalakas A.M., Kirchner H.L., Grewal H.M., Schaaf H.S., van Wyk S.S., Hesseling A.C. (2011). Environmental tobacco smoke exposure increases *Mycobacterium tuberculosis* infection risk in children. Int. J. Tuberc. Lung Dis..

[57] Lindsay R.P., Shin S.S., Garfein R.S., Rusch M.L., Novotny T.E. (2014). The Association between active and passive smoking and latent tuberculosis infection in adults and children in the united states: Results from NHANES. PLoS ONE.

[58] Ghio A.J., Soukup J.M., Dailey L.A., Madden M.C. (2020). Air pollutants disrupt iron homeostasis to impact oxidant generation, biological effects, and tissue injury. Free Radic. Biol. Med..

[59] Reemtsma T., These A., Venkatachari P., Xia X., Hopke P.K., Springer A., Linscheid M. (2006). Identification of fulvic acids and sulfated and nitrated analogues in atmospheric aerosol by electrospray ionization fourier transform ion cyclotron resonance mass spectrometry. Anal. Chem..

[60] Dimala C.A., Kadia B.M. (2022). A systematic review and meta-analysis on the association between ambient air pollution and pulmonary tuberculosis. Sci. Rep..

[61] Mao J.J., Chen H.L., Li C.H., Lu J.W., Gu Y.Y., Feng J., Zhang B., Ma J.F., Qin G. (2023). Population impact of fine particulate matter on tuberculosis risk in China: A causal inference. BMC Public Health.

[62] Wang S., Wu G., Du Z., Wu W., Ju X., Yimaer W., Chen S., Zhang Y., Li J., Zhang W. (2023). The causal links between long-term exposure to major PM2.5 components and the burden of tuberculosis in China. Sci. Total Environ..

[63] Jassal M.S., Bakman I., Jones B. (2013). Correlation of ambient pollution levels and heavily-trafficked roadway proximity on the prevalence of smear-positive tuberculosis. Public Health.

[64] Linde L.R., Readhead A., Barry P.M., Balmes J.R., Lewnard J.A. (2023). Tuberculosis Diagnoses Following Wildfire Smoke Exposure in California. Am. J. Respir. Crit. Care Med..

[65] García-Sancho M.C., García-García L., Báez-Saldaña R., Ponce-De-León A., Sifuentes-Osornio J., Bobadilla-Del-valle M., Ferreyra-Reyes L., Cano-Arellano B., Canizales-Quintero S., Palacios-Merino Ldel C. (2009). Indoor pollution as an occupational risk factor for tuberculosis among women: A population-based, gender oriented, case-control study in Southern Mexico. Rev. Investig. Clin..

[66] Mishra V.K., Retherford R.D., Smith K.R. (1999). Biomass cooking fuels and prevalence of tuberculosis in India. Int. J. Infect. Dis..

[67] Mills P.K., Beaumont J.J., Nasseri K. (2006). Proportionate mortality among current and former members of the United Farm Workers of America, AFL-CIO, in California 1973–2000. J. Agromed..

[68] Dugger D.L., Stanton S.H., Irby B.N., McConnell B.L., Cummings W.W., Mattman R.W. (1964). The exchange of twenty metal ions with the weakly acidic silanol group of silica gel. J. Phys. Chem..

[69] Jenkins P.A. (1981). The epidemiology of opportunist mycobacterial infections in Wales, 1952–1978. Rev. Infect. Dis..

[70] Ehrlich R., Murray J., Said-Hartley Q., Rees D. (2024). Silicotuberculosis: A critical narrative review. Eur. Respir. Rev..

[71] Howlett P., Said B., Mwanga E., Mbuya A., Nota M., Kon O.M., Gottesfeld P., Feary J., Mpagama S., Ehrlich R. (2025). Confronting the growing epidemic of silicosis and tuberculosis among small-scale miners. Lancet Public Health.

[72] Wood W.B., Gloyne S.R. (1931). Pulmonary asbestosis complicated by pulmonary tuberculosis. Lancet.

[73] Sheers G. (1964). Prevalence of pneumoconiosis in Cornish kaolin workers. Br. J. Ind. Med..

[74] Devilliers A.J., Windish J.P. (1964). Lung cancer in a fluorspar mining community. I. Radiation, dust, and mortality experience. Br. J. Ind. Med..

[75] Dias O.M., Canzian M., Terra-Filho M., Santos Ude P. (2011). Talc asbestosis and pulmonary tuberculosis in a patient exposed to the talc used in the production of soccer balls. J. Bras. Pneumol..

[76] Wang Y., Wang R., Ming J., Liu G., Chen T., Liu X., Liu H., Zhen Y., Cheng G. (2016). Effects of dust storm events on weekly clinic visits related to pulmonary tuberculosis disease in Minqin, China. Atmos. Environ..

[77] Roviaro G.C., Sartori F., Calabrò F., Varoli F. (1982). The association of pleural mesothelioma and tuberculosis. Am. Rev. Respir. Dis..

[78] De Coster C., Verstraeten J.M., Dumortier P., De Vuyst P. (1996). Atypical mycobacteriosis as a complication of talc pneumoconiosis. Eur. Respir. J..

[79] Sonnenberg P., Murray J., Glynn J.R., Thomas R.G., Godfrey-Faussett P., Shearer S. (2000). Risk factors for pulmonary disease due to culture-positive, *M. tuberculosis* or nontuberculous mycobacteria in South African gold miners. Eur. Respir. J..

[80] Izhakian S., Slobodscoy Ignatov L., Gorenshtein A., Rothschild B., Fireman E., Rosengarten D., Kramer M.R. (2025). Non-tuberculous Mycobacterium Infection Associated with Artificial Stone Silicosis: A Case Series. Isr. Med. Assoc. J..

[81] Tremblay G.A. (2007). Historical statistics support a hypothesis linking tuberculosis and air pollution caused by coal. Int. J. Tuberc. Lung Dis..

